# Vertical Transmission of Avian Bornavirus in Psittacines

**DOI:** 10.3201/eid1712.111317

**Published:** 2011-12

**Authors:** Michael Lierz, Anne Piepenbring, Christiane Herden, Kirstin Oberhäuser, Ursula Heffels-Redmann, Dirk Enderlein

**Affiliations:** Justus Liebig University, Giessen, Germany (M. Lierz, A. Piepenbring, C. Herden, U. Heffels-Redmann, D. Enderlein);; Loro Parque Fundacion, Tenerife, Spain (K. Oberhauser)

**Keywords:** proventricular dilatation disease, species conservation, birds, psittacines, avian, parrots, embryo, hatching, viruses

**To the Editor**: Proventricular dilatation disease (PDD) is a fatal disease in psittacines that jeopardizes critical species conservation projects, such as that involving the Spix’s macaw (*Cyanopsitta spixii*), the world’s most endangered bird species (*1*). The disease is characterized by lymphoplasmacytic infiltrations in the enteric and central nervous systems (*2*). Consequently, gastrointestinal and neurologic disorders are the major clinical manifestations. Only recently has the cause of the disease been identified by characterization of a newly discovered member of the family *Bornaviridae*, the avian bornavirus (ABV), which has been detected in affected psittacines (*3**,**4*). The relationship of an infection with ABV and the occurrence of PDD has been described in natural cases (*5**,**6*) and in experimental trials (*7**,**8*). However, birds that are infected with ABV but that are clinically healthy have also been recognized (*6*). Infected birds can shed viral RNA intermittently (*9*), and not all infected birds seroconvert (*5*). For psittacine flock management, control of an AΒV infection is critical, e.g., by repeated testing of breeding stock and removal of ABV-positive birds (*2**,**5*). However, in breeding projects of rare species, every individual is genetically important and cannot be lost. Therefore, pairing infected, but clinically healthy, birds separately from birds that test negative for the virus might represent an option. For this possibility to be viable, whether vertical transmission of ABV can take place must be further clarified. A study investigating the distribution of ABV in tissues of PDD-positive birds has demonstrated ABV antigen in follicular cells, which may point toward vertical transmission (*9*).

To investigate vertical transmission of ABV, we examined 30 dead-in-shell embryos of various psittacine species that originated from ABV-infected flocks with a history of PDD. First, the eggshell was disinfected and opened at the blunt end by using sterile equipment. The brain and proventriculus of each embryo were analyzed for the presence of ABV RNA by using 2 different real-time reverse transcription PCRs, as described by Honkavuori (*4*), with the primer pair 1034–1322 and, in case of a negative result, the additional primer set 1367. Sampling, RNA extraction, and PCR were repeated by using the same methods to exclude possible cross-contamination of samples. Afterwards, the complete embryo was placed in 10% buffered formalin, and histopathologic examination and immunohistochemical (IHC) testing were carried out (*10*) with antibodies directed against the viral phosphoprotein and X protein. If ABV RNA or ABV antigen was demonstrated, crop and cloacal swab specimens and serum of the parents of the positive embryo were immediately taken and used either for ABV RNA detection with the above described PCR or for the detection of specific ABV antibodies by indirect immunofluorescence assay (*10*). This procedure was chosen because earlier sampling of the parents might have caused breeding interruption, and which eggs of which parents would be available for investigation was not clear.

In 2 of the 30 embryos investigated, ABV RNA was detected by using the 1034 PCR. The repeated procedure provided the same results in the same embryos. One embryo was a Major Mitchell cockatoo (*Cacatua leadbeateri*) (cycle threshold 31.41) and the other a red-crowned Amazon (*Amazona viridigenalis*) (cycle threshold 35.1). None of the investigated embryos demonstrated histopathologic lesions typical of an ABVinfection. IHC testing did not show any positive results. However, in the ABV-positive Amazon embryo, an equivocal result was obtained.

The swab specimens of both parents of the Major Mitchell cockatoo tested positive for ABV RNA, but serum did not demonstrate specific ABV antibodies. The crop swab specimen of the female red crowned Amazon was positive for ABV RNA, but serum was negative for ABV antibodies; the male bird tested negative by PCR but demonstrated an ABV-specific antibody titer of 80.

These results highlight the potential risk for vertical transmission of ABV and the conclusion that ABV-infected parents can most likely produce infected offspring. However, test results were positive for only 2 of 30 eggs. Because potentially dead-in-shell embryos are usually further incubated by the breeder to ensure embryonic death, the possibility cannot be excluded that ABV RNA was already degraded in some cases, thus causing false-negative results. On the other hand, these eggs might have originated from ABV-negative parents. The quality of the samples might also have caused the questionable IHC results. In PDD-affected, ABV-positive flocks >30% of the birds could be infected (*5**,**6*) and the virus is shed intermittently (*9*). Therefore, pairing ABV-positive birds, incubating their eggs artificially, and raising the chicks separately until they show negative test results, might be an option for breeding projects. However, when vertical transmission occurs (and, if so, its incidence) is unknown.

Whether ABV infection of the embryos was the cause of death remains unclear. Even if typical lesions were not detected, the poor quality of the material might have hidden such lesions. However, embryonic infection that does not result in embryonic death is the basic requirement for successful vertical transmission. These preliminary results warrant further studies investigating the possibility of vertical transmission by ABV-infected pairs, especially to minimize the risk for such transmission to endangered species with restricted breeding opportunities.
